# Supplementary material for: A hierarchical cellular structural model to unravel the universal power-law rheological behavior of living cells
Source: Nat Commun. 2021 Oct 18;12:6067. doi: 10.1038/s41467-021-26283-y (PMC8523554; doi:10.1038/s41467-021-26283-y)
Supplement: Supplementary file 1 — Supplementary Information [file 41467_2021_26283_MOESM1_ESM.pdf]

Supplementary Notes for "A hierarchical cellular structural model to unravel the universal power-law rheological behavior of living cells"

Jiu-Tao Hang,[1] Yu Kang,[2] Guang-Kui Xu,[1*] and Huajian Gao[3,4*]

[1] Laboratory for Multiscale Mechanics and Medical Science, Department of Engineering Mechanics, SVL, School of Aerospace Engineering, Xi'an Jiaotong University, Xi'an 710049, China
[2] College of Pharmaceutical Sciences, Zhejiang University, Hangzhou 310058, China
[3] School of Mechanical and Aerospace Engineering, College of Engineering, Nanyang Technological University, Singapore 639798, Singapore
*[4] Institute of High Performance Computing, A*STAR, Singapore 138632, Singapore*

Supplementary Note 1. Creep compliance of Kelvin-Voigt material in 3D

The creep response of the Kelvin-Voigt model is:

$$J_{\mathrm{KV}}(t) = \left(1 - e^{-t/\tau}\right)/E\,, \tag{S1}$$

where J_{KV} is the compliance, t the time, E the elastic modulus, and τ relaxation time. When $t/\tau \ll 1$, it takes a power-law form with an exponent of 1.0. The creep response of Kelvin-Voigt material in 3D can be expressed as:

$$\begin{aligned} &J_x(t) = C\left(1 - ae^{-\lambda t}\right),\ a = \frac{2(1+\mu)}{3},\ C = \frac{1}{9K} + \frac{2(1+\mu)}{9K(1-2\mu)} \\ &J_y(t) = J_z(t) = C'\left(1 - a'e^{-\lambda t}\right),\ a' = \frac{1+\mu}{3\mu},\ C' = \frac{1+\mu}{9K(1-2\mu)} - \frac{1}{9K},\ C' < 0 \end{aligned}\ . \tag{S2}$$

Because of the transverse elastic expansion, the creep response of the material is equivalent to the standard linear viscoelastic model that consists of Kelvin-Voigt models and springs in series. As shown in Supplementary Fig. 1, if the cytoskeleton is neglected, the displacement response of cells shows a power-law dependence on time, even if the cytoplasm takes different Poisson's ratios. The power-law exponents are

*Corresponding authors.
E-mail: guangkuixu@mail.xjtu.edu.cn or huajian.gao@ntu.edu.sg

approximately 0.5, which is the upper limit of scale-free cell rheology [1].

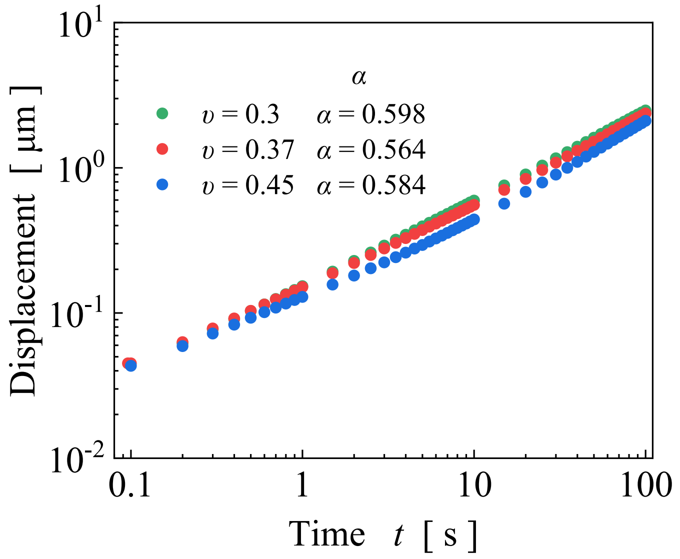

Supplementary Fig. 1 Creep responses of the adopted cell model without cytoskeleton for the cytoplasm with different Poisson's ratios.

Supplementary Note 2. Creep compliance of the self-similar hierarchical model over time

Schiessel and Blumen [2] obtained solutions to the complex modulus of the ladder viscoelastic model by using a method of continued fractions, which is much complicated and not suitable for expansion. Here we propose a simple yet robust method to obtain the creep compliance of our self-similar hierarchical model over time. The creep compliances of 1st, 2nd, and 3rd level hierarchies are denoted by J_1, J_2 and J_3, respectively. Taking J_1 as an example, the serial number k of each element in the 1st level hierarchy increases from left to right and from bottom to up, as shown in Supplementary Fig. 2a.

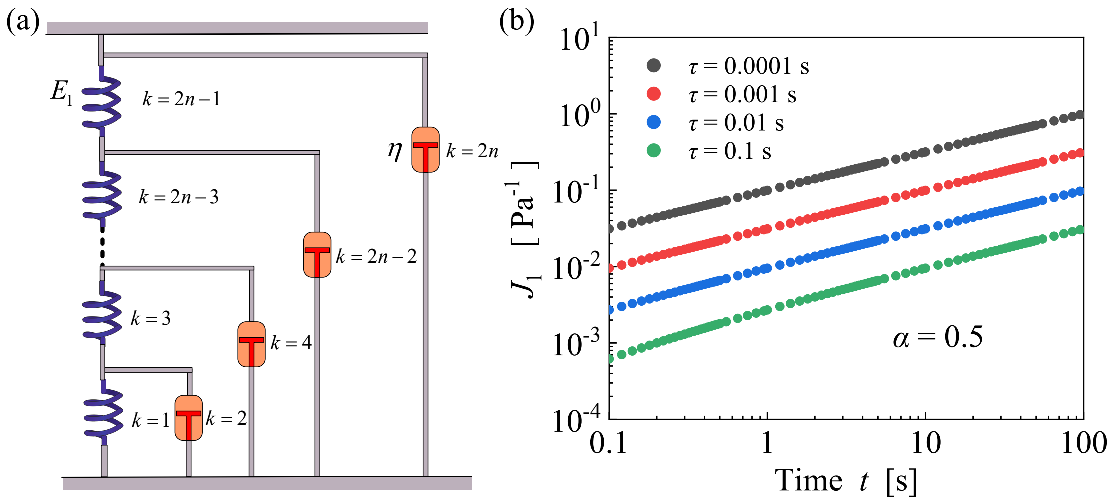

Supplementary Fig. 2 (a) Schematic diagram of the 1st level hierarchy. (b) Creep compliances of the 1st level hierarchy model for different values of τ .

The recurrence relations in numerical analysis are:

$$\begin{aligned} J_1(1) &= \frac{1}{E_1} \\ J_1(2) &= \frac{1}{1/J_1(1)+\eta_1/t} \\ &\vdots \\ J_1(2n-1) &= J_1(2n-2)+\frac{1}{E_1} \\ J_1(2n) &= \frac{1}{1/J_1(2n-1)+\eta_1/t} \end{aligned} . \tag{S3}$$

Thus, the relation between $J_1(2n-2)$ and $J_1(2n)$ is $J_1(2n) = 1/\left(\left(J_1(2n-2)+1/E_1\right)+\eta_1/t\right)$. When n tends to be infinite, one has $J_1(2n-2) = J_1(2n)$. Consequently, the creep compliance of the 1st level hierarchy is simplified to be:

$$\eta E_1 J_1^2 + \eta J_1 - t = 0 , \tag{S4}$$

for which the solution is:

$$J_1 = \frac{1}{2E_1}\left(-1+\sqrt{1+\frac{4t}{\tau}}\right). \tag{S5}$$

Since the elastic modulus of the cytoplasm is in the range of $10^2 \sim 10^4$ Pa [3] and the viscosity of the cytoplasm is in the range of $10^{-3} \sim 10^1$ Pa•s [4–7], one can evaluate τ in the range of $10^{-4} \sim 10^{-1}$ s. Comparing with the time in experiments [3–7], the value of τ is sufficiently small. Therefore, the creep compliance of the 1st level hierarchy approaches:

$$\lim_{t/\tau\to\infty} J_1 = \lim_{t/\tau\to\infty} \frac{-1+(1+4t/\tau)^{0.5}}{2E_1} = \frac{(t/\tau)^{0.5}}{E_1} \propto t^{0.5}. \tag{S6}$$

Considering a cell without cytoskeleton, the self-similar hierarchical model will reduce to the 1st level hierarchy. Supplementary Fig. 2b shows that the power-law exponent of the 1st level hierarchy is approximately 0.5 under different values of τ, in agreement with Eq. (S6) and our simulation results in Supplementary Fig. 1.

Analogously, we can obtain the analytic expressions of creep compliances of the 2nd and 3rd level hierarchies:

$$J_2 = \frac{-1+\sqrt{1+4E_2J_1}}{2E_2}, \tag{S7}$$

$$J_3 = \frac{-1+\sqrt{1+4E_3J_2}}{2E_3}. \tag{S8}$$

If $E_2J_1 \gg 1$, J_2 can be rewritten as:

$$J_2 = \frac{-1+\sqrt{1+4E_2J_1}}{2E_2} \approx \frac{\sqrt{E_2J_1}}{E_2} \propto \sqrt{J_1}. \qquad \text{(S9).}$$

The power-law exponent of J_2 is half of that of J_1. If $E_2J_1 \ll 1$, J_2 is re-expressed as:

$$J_2 = \frac{-1+\sqrt{1+4E_2J_1}}{2E_2} \approx \frac{2E_2J_1}{2E_2} \propto J_1. \qquad \text{(S10).}$$

In this case, J_2 has the same power-law exponent as J_1. Hence, the power-law exponent α of J_2 falls in the range of 0.25 ~ 0.5.

When $E_3 J_2 \gg 1$, J_3 can be rewritten as:

$$J_3 = \frac{-1+\sqrt{1+4E_3J_2}}{2E_3} \approx \frac{\sqrt{E_3J_2}}{E_3} \propto \sqrt{J_2}\,. \tag{S11}$$

If $E_3 J_2 \ll 1$, J_3 is given by:

$$J_3 = \frac{-1+\sqrt{1+4E_3J_2}}{2E_3} \approx \frac{2E_3J_2}{2E_3} \propto J_2\,. \tag{S12}$$

Similar to the above analyses, the power-law exponent α of J_3 is in the range of 0.125 ~ 0.5. To further confirm these findings, we have performed a series of numerical simulations, as shown in Supplementary Fig. 3. It can be seen that the power-law exponents of 2nd and 3rd level hierarchies are in the range of 0.25 ~ 0.5 (Supplementary Fig. 3a) and 0.125 ~ 0.5 (Supplementary Fig. 3b), respectively. For each level of the self-similar hierarchical model, α decreases gradually with the increase of the spring stiffness (E_2 or E_3). The minimal power-law exponent of each level is half of that of its lower level. The power-law exponent of our model falls in the range of 0.125 ~ 0.5, which is in agreement with a vast variety of experiments [1]. Since the elastic stiffness of MT is much higher than that of the cytoplasm and E_2 is close to E_3, we conjecture that the power-law exponent will be concentrated around 0.25, as found in experiments [8, 9].

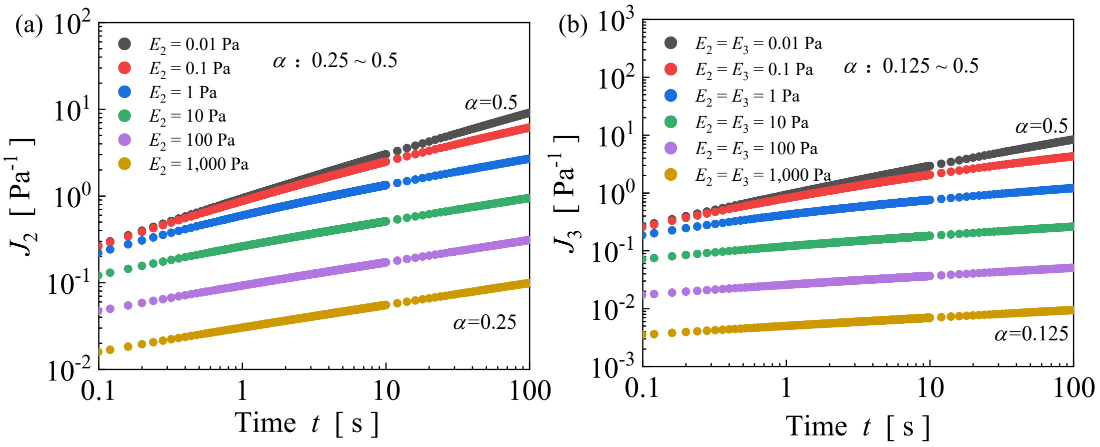

Supplementary Fig. 3 The power-law rheological responses of the (a) 2nd and (b) 3rd level

hierarchies by varying parameters. Here we take $\eta = 0.1$ Pa•s and $E_1 = 10$ Pa .

Supplementary Note 3. Complex modulus of the self-similar hierarchical model over frequency

Here we propose a method to obtain the storage modulus E' and the loss modulus E'' of the self-similar hierarchical model over frequency. The complex modulus of the spring is its elastic modulus, while the complex modulus of the dashpot is $i\omega\eta$ with ω being angular frequency. Let G_1, G_2 and G_3 denote the complex moduli of 1st, 2nd, and 3rd level hierarchies, respectively. Similar to the above section, the recurrence relations of the 1st level hierarchy in numerical analysis are:

$$\begin{aligned} &G_1(1) = E_1 \\ &G_1(2) = E_1 + i\omega\eta \\ &\quad\vdots \\ &G_1(2n-1) = 1/\left(1/G_1(2n-2) + 1/E_1\right) \\ &G_1(2n) = G_1(2n-1) + i\omega\eta \end{aligned} . \tag{S13}$$

When n tends to be infinite, $G_1(2n-2) = G_1(2n)$. Consequently, the complex modulus of the 1st level hierarchy is simplified as:

$$G_1^2 - i\omega\eta G_1 - i\omega\eta E_1 = 0, \qquad \text{(S14).}$$

for which the solution is:

$$G_1 = \frac{i\omega\eta + \sqrt{(i\omega\eta)^2 + 4i\omega\eta E_1}}{2}. \qquad \text{(S15).}$$

Using $\tau = \eta/E_1$ to replace η, one can get:

$$G_1 = E_1 \frac{1 + \sqrt{1 + 4(i\omega\tau)^{-1}}}{2(i\omega\tau)^{-1}}. \qquad \text{(S16).}$$

Analogously, we can obtain the analytic expressions of complex moduli of 2nd and 3rd level hierarchies:

$$G_2 = \frac{G_1 + \sqrt{(G_1)^2 + 4E_2G_1}}{2}, \quad \text{(S17).}$$

$$G_3 = \frac{G_2 + \sqrt{(G_2)^2 + 4E_3G_2}}{2}. \quad \text{(S18).}$$

It can be seen from Eq. (S16) that with increasing frequency, the loss modulus increases faster than the storage modulus, because the term $i\omega\tau$ increases the proportion of the imaginary part of the complex modulus. Thus, the power-law exponent of the loss modulus will be larger than that of the storage modulus at high frequencies, which is confirmed in experiments [7, 10] and our simulation results (Fig. 1b).

Supplementary Note 4. Stress stiffening behavior of cells

Many biological systems, including cells, exhibit stress stiffening behavior under static loadings, which reflects the strong nonlinearity of their stress-strain relations. In the cortex layer, there are abundant microtubules (MTs), intermediate filaments (IFs), microfilaments (MFs), and binding proteins [11]. This layer can be modeled by a prismatic tensegrity (Supplementary Fig. 4) in which rods and strings are used to represent components bearing compressive and tensile loads, respectively, since the tensegrity structure model can well describe the mechanical response of cells [12, 13]. In the modeling, the microfilaments and intermediate filaments in the cytoskeleton are treated as tension strings, and the microtubules are considered as compression bars. The prismatic tensegrity structure consists of top strings, bottom strings, diagonal strings, and bars [14]. Supplementary Fig. 4 shows some vertices and elements in a v-prismatic tensegrity. Here, v is the number of sides of the polygon. As shown in Supplementary Fig. 1, a is the number of torsional sides of the top and bottom polygons that satisfies $1 \le a < \upsilon$.

r_1 and r_2 are the radii of the top and bottom circles, respectively. Let β denote the relative torsion angle between the top and bottom polygons, and q_{s1}, q_{s2}, q_{s3} and q_b represent the force density of top strings, bottom strings, diagonal strings, and bars, respectively. Then, the analytic solution for the equilibrium configuration of such a *v*-prismatic structure can be expressed as [15]:

$$\frac{\beta}{\pi}=\frac{1}{2}-\frac{a}{v}$$
$$-\frac{q_{s1}}{q_b}=\frac{r_2\sin\frac{\pi a}{v}}{r_1\left(1-\cos\frac{2\pi}{v}\right)} \qquad -\frac{q_{s2}}{q_b}=\frac{r_1\sin\frac{\pi a}{v}}{r_2\left(1-\cos\frac{2\pi}{v}\right)} \qquad -\frac{q_{s3}}{q_b}=1 . \tag{S19}$$

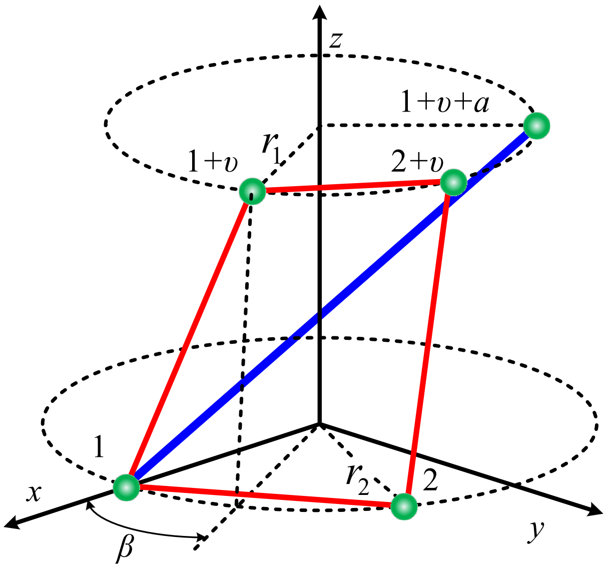

Supplementary Fig. 4 Elements meeting at node 1 in a *v*-prismatic tensegrity.

Here, we use a combination of the cortex layer and emanative MTs with different lengths as the cytoskeleton (see Inset of Supplementary Fig. 5). MFs are solid truss elements with a cross-section of $20\ \mathrm{nm}^2$ and modulus of 2400 MPa [16]. Experiments [17, 18] showed that the differential stiffness E_d of cells increases linearly with the externally applied stress σ, i.e., $E_d = d\sigma/d\varepsilon = E_0 + \lambda\sigma$ where λ is the stress stiffening factor. Accordingly, one can obtain the stress-strain relation

$\sigma = E_0(e^{\lambda\varepsilon} - 1)/\lambda$. To examine whether our model can capture this behavior, we apply static loads on the cell and plot the stress-strain curves with different cortex layer structures in Supplementary Fig. 5a. It can be seen that the curves can be well fitted by the function $\sigma = E_0(e^{\lambda\varepsilon} - 1)/\lambda$, indicating that our model can also simulate the stress stiffening behavior of cells. The stiffening mainly originates from the structural configurational change of the cytoskeleton with cytoskeleton filaments gradually rotating to align with the loading direction during the deformation process. Prestress in living cells is a key regulator of many cellular functions [19]. By varying the prestrain of the strings in the tensegrity, we can regulate the prestress level in the cell. We find that the cellular stiffness calculated from this model is proportional to the prestrain (Supplementary Fig. 5b), which agrees with relevant experimental results [19].

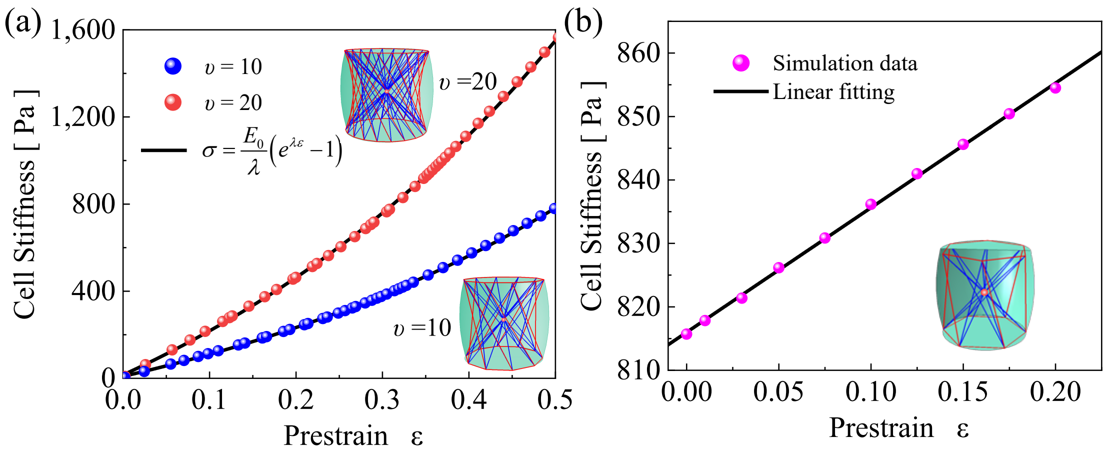

Supplementary Fig. 5 Predicted stress stiffening behavior from the cellular tensegrity model. (a) The stress-strain curves in the cases of 10-prismatic and 20-prismatic tensegrity structures. The simulation data can be well fitted by the function $\sigma = E_0(e^{\lambda\varepsilon} - 1)/\lambda$. (b) The cellular stiffness increases linearly with prestrain.

Supplementary Reference

1. Kollmannsberger, P. & Fabry, B. Linear and nonlinear rheology of living cells. *Annu. Rev. Mater. Res.* **41,** 75–97 (2011).

2. Schiessel, H. & Blumen, A. Mesoscopic Pictures of the Sol-Gel Transition: Ladder Models and Fractal Networks. *Macromolecules.* **28,** 4013 (1995).

3. Levental, K. *et al.* Matrix Crosslinking Forces Tumor Progression by Enhancing Integrin Signaling. *Cell.* **139,** 891–906 (2009).

4. Emad, M. *et al.* The cytoplasm of living cells behaves as a poroelastic material. *Nat. Mater.* **12,** 253–261 (2013).

5. Panhwar, M. H. *et al.* High-throughput cell and spheroid mechanics in virtual fluidic channels. *Nat. Commun.* **11,** 2190 (2020).

6. Zhou, E. *et al.* Universal behavior of the osmotically compressed cell and its analogy to the colloidal glass transition. *Proc. Natl. Acad. Sci. USA.* **106,** 10632–10637 (2009).

7. Fabry, B. *et al.* Scaling the microrheology of living cells. *Phys. Rev. Lett.* **87,** 148102 (2001).

8. Delphine, I. A., Olivier, C., Alain, R. & Sylvie, H. Cell stiffening in response to external stress is correlated to actin recruitment. *Biophys. J.* **94,** 2906–2913 (2008).

9. Nicolas, D., Alain, R., Jacqueline, S. & Atef, A. Creep function of a single living cell. *Biophys. J.* **88,** 2224–2233 (2005).

10. Rigato, A., Miyagi, A., Scheuring, S. & Rico, F. High-frequency microrheology reveals cytoskeleton dynamics in living cells. *Nat. Phys.* **13,** 771–775 (2017).

11. Karp, G. *Cell and molecular biology: concepts and experiments*. John Wiley & Sons, 2009.

12. Kardas, D., Nackenhorst, U. & Balzani, D. Computational model for the cell-mechanical response of the osteocyte cytoskeleton based on self-stabilizing tensegrity structures. Biomechanics and Modeling in Mechanobiology 12, 167-

183 (2013).

13. Wang, N. et al. Mechanical behavior in living cells consistent with the tensegrity model. Proc. Natl. Acad. Sci. USA 98, 7765-7770 (2001).

14. Zhang, L. Y. *et al.* A Numerical Method for Simulating Nonlinear Mechanical Responses of Tensegrity Structures Under Large Deformations. *J. Appl. Mech.* **80,** 061018

15. Murakami, H. Static and dynamic analyses of tensegrity structures. Part II. Quasi-static analysis. *Int. J. Solids. Struct.* **38,** 3615–3629 (2001).

16. Gittes, F., Mickey, B., Nettleton, J. & Howard, J. Flexural rigidity of microtubules and actin filaments measured from thermal fluctuations in shape. J. Cell. Biol. 120, 923–934 (1993).

17. Wendling, S., Oddou, C. & Isabey, D. Stiffening response of a cellular tensegrity model. *J. Theor. Biol.* **196,** 309 (1999).

18. Wang, N., Butler, J. P. & Ingber, D. E. Mechanotransduction across the cell surface and through the cytoskeleton. *Science* **260,** 1124–1127 (1993).

19. Wang, N. *et al.* Mechanical behavior in living cells consistent with the tensegrity model. *Proc. Natl. Acad. Sci. USA.* **98,** 7765–7770 (2001).
