# Supplementary material for: A hierarchical cellular structural model to unravel the universal power-law rheological behavior of living cells
Source: Nat Commun. 2021 Oct 18;12:6067. doi: 10.1038/s41467-021-26283-y (PMC8523554; doi:10.1038/s41467-021-26283-y)
Supplement: Supplementary file 3 — Description of Additional Supplementary Files [file 41467_2021_26283_MOESM3_ESM.pdf]

Description of Additional Supplementary Files

File Name: Supplementary Software 1
Description: The folder contains three files:
The ModelScripts.py is the code file for modelling, which is suitable for running on Abaqus 6.13-1.
The readme.txt file gives the instructions for running the code.
The Parameters In the Model.pptx gives the physical meaning of the parameters those are entered in the code.
