# Supplementary figures and images for: A hierarchical cellular structural model to unravel the universal power-law rheological behavior of living cells
Source: Nat Commun. 2021 Oct 18;12:6067. doi: 10.1038/s41467-021-26283-y (PMC8523554; doi:10.1038/s41467-021-26283-y)

## Slide 1
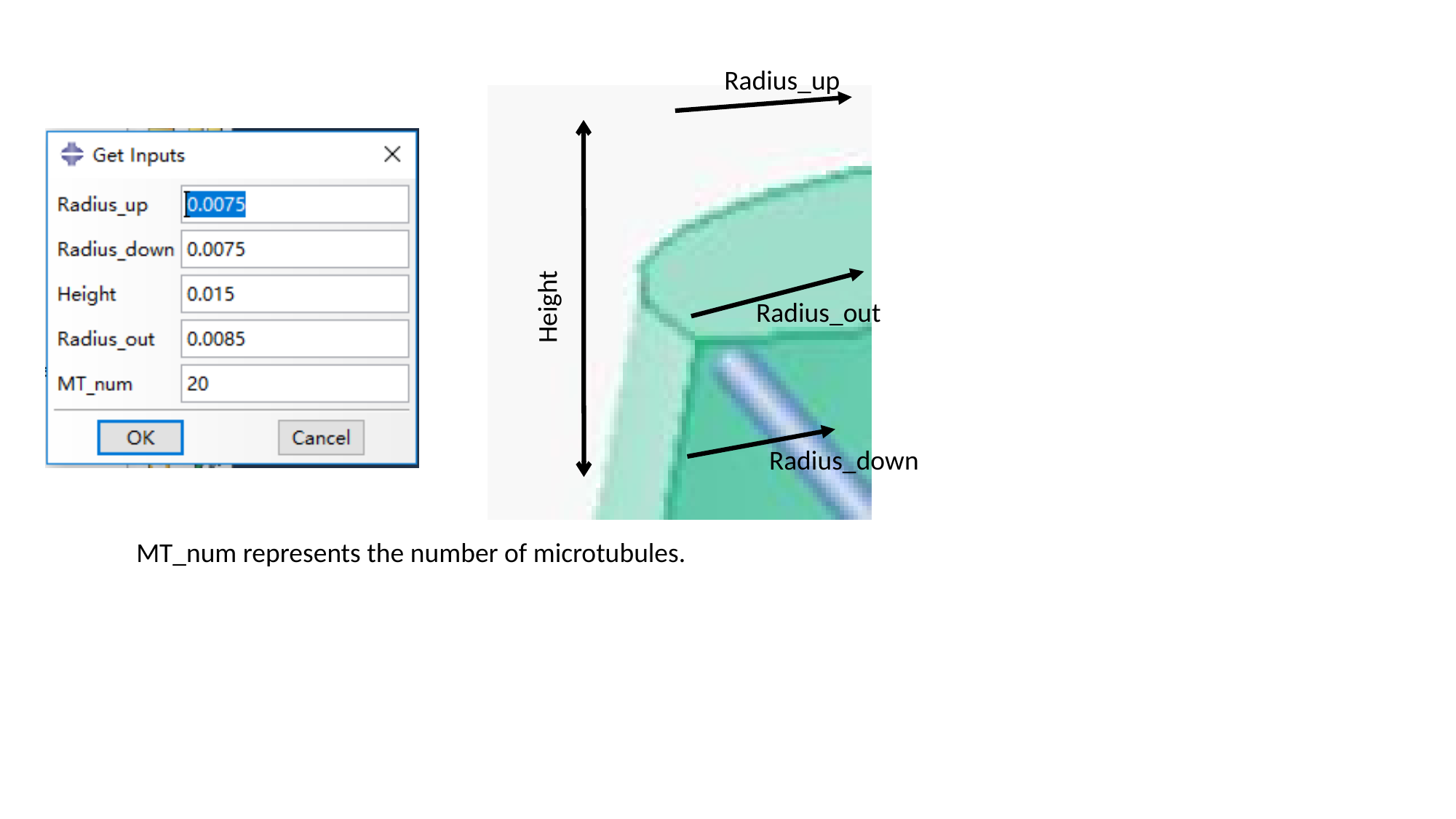

Radius_up
Height
Radius_out
Radius_down
MT_num represents the number of microtubules.

Supplement: Supplementary file 4 — Supplementary Software 1 [file 41467_2021_26283_MOESM4_ESM.zip › Supplementary Software 1/Parameters In the Model.pptx]
